# Regulation of nicotinic acetylcholine receptor turnover by MuRF1 connects muscle activity to endo/lysosomal and atrophy pathways

**DOI:** 10.1007/s11357-012-9468-9

**Published:** 2012-09-06

**Authors:** Rüdiger Rudolf, Julius Bogomolovas, Siegfried Strack, Kyeong-Rok Choi, Muzamil Majid Khan, Anika Wagner, Kathrin Brohm, Akira Hanashima, Alexander Gasch, Dittmar Labeit, Siegfried Labeit

**Affiliations:** 1 Institute of Toxicology and Genetics, Karlsruhe Institute of Technology, Hermann-von-Helmholtz-Platz 1, 76344 Eggenstein-Leopoldshafen, Germany; 2 Institute of Molecular and Cell Biology, University of Applied Sciences Mannheim, Windeckstrasse 110, 68163 Mannheim, Germany; 3 Institute of Medical Technology, University of Heidelberg and University of Applied Sciences Mannheim, Paul-Wittsack-Strasse 10, 68163 Mannheim, Germany; 4 Department for Integrative Pathophysiology, Universitätsmedizin Mannheim, Theodor-Kutzer-Ufer, 68167 Mannheim, Germany

**Keywords:** Acetylcholine receptor, Atrophy, Endocytosis, MuRF1, Neuromuscular junction

## Abstract

Muscle atrophy is a process of muscle wasting induced under a series of catabolic stress conditions, such as denervation, disuse, cancer cachexia, heart and renal failure, AIDS, and aging. Neuromuscular junctions (NMJs), the synapses between motor neurons and muscle fibers undergo major changes in atrophying muscles, ranging from mild morphological alterations to complete disintegration. In this study, we hypothesized that remodeling of NMJs and muscle atrophy could be linked together. To test this, we examined if a major atrophy-promoting E3 ubiquitin ligase, MuRF1, is involved in the maintenance of NMJs. Immunofluorescence revealed that MuRF1 is highly enriched close to the NMJ. Affinity precipitation and in vivo imaging showed that MuRF1 interacts in endocytic structures with both, acetylcholine receptor, the primary postsynaptic protein of the NMJ, as well as with Bif-1, an autophagy- and endocytosis-regulating factor. In vivo imaging, radio labeling, and weighing approaches demonstrated that metabolic destabilization of acetylcholine receptors and muscle atrophy induced by denervation were significantly rescued in MuRF1-KO animals. Notably, interaction with Bif-1, and the rescue of AChR lifetime and muscle atrophy were specific to MuRF1 but not MuRF2. Our data demonstrate an involvement of MuRF1 in membrane protein-turnover, including the degradation of AChRs at the NMJ under atrophying conditions where MuRF1 also interacts and associates with Bif-1.

## Introduction

Progressive reduction of skeletal muscle mass and contractility increasingly represents a major health issue in the aging populations of developed countries. Major causes for muscle wasting include sedentary lifestyle, a series of diseases — such as diabetes, cancer, AIDS, heart and renal failure — and aging itself. Although muscle atrophy is usually considered as a primary disorder of the sarcomere, there are intimate connections between innervation activity and atrophy: For example, denervation (sciatic nerve lesion) is used in rodents to induce atrophy experimentally (Bodine et al. 2001; Cohen et al. 2009), and in the clinic therapeutic blockade of the neuromuscular junction (NMJ) by depolarizing agents is a risk factor for aging-related atrophy in critical illness myopathy (Latronico et al. 2005a, b). Furthermore, NMJ gross morphology significantly deteriorates in aging muscle resulting in progressive fragmentation of synapses and partial functional denervation (Tuffery 1971; Gutmann and Hanzlikova 1976; Pestronk et al. 1980; Courtney and Steinbach 1981; Valdez et al. 2010; Chen et al. 2012). Of note, genetically induced NMJ fragmentation recapitulates major aspects of aging-related atrophy, such as fiber type grouping, fiber size heterogeneity, and fiber loss (Butikofer et al. 2011). Finally, typical atrophy treatments used in clinics such as resistance training and metabolic manipulations reverse the severe fragmentation of aging NMJs (Andonian and Fahim, 1987, 1988; Valdez et al. 2010). On the molecular level, both denervation and NMJ blockade activate the so-called atrogene gene program (Bodine et al. 2001; Lecker et al. 2004; Sandri et al. 2004). This program includes, in particular, a family of E3 ubiquitin ligases that are considered as central players in the degradation of muscle proteins (Bodine et al. 2001; Lecker et al. 2004). Prompted by this apparent convergence of mechanisms in muscle atrophy and NMJ deformation we here investigated the role of the classical muscle atrophy marker, MuRF1, in the maintenance of the NMJ. Our data suggest that atrophy and functional denervation are intimately connected and that they are two aspects of the same adaptive chronic stress process, which should be considered together when developing treatment strategies.

## Methods

### Animals

The MuRF1−/− KO and MuRF2−/− KO lines used here have been described previously (Witt et al. 2008). Here, 10- to 14-week-old MuRF1 and MuRF1 KO mice were backcrossed for ten generations on a C57BL/6 J background. Animals were maintained in the local animal facilities. Use and care of animals were as approved by German authorities and according to national law (TierSchG §§7). Anaesthesia was administered using either inhalation of Isoflurane (cp-pharma) or intraperitoneal (i.p.) injection of Rompun (Bayer) and Zoletil 100 (Laboratoires Virbac). For hindlimb denervation, 5 mm of the sciatic nerve were removed. Success of denervation was checked in sacrificed mice at the end of each experiment. For transfection of isolated mouse models, we electroporated expression plasmids essentially as previously described (Dona et al. 2003; Choi et al. 2012; Rudolf et al. 2012).

### Immunofluorescence

For immunofluorescence, 15-μm-thick transversal sections were prepared from snap-frozen extensor digitorum longus muscles. Stainings used α-bungarotoxin (BGT) fluorescently labeled with AlexaFluor 555 (BGT-AF555) to mark acetylcholine receptors (AChRs) in NMJs. MuRF1 was detected with three different antibodies as described previously with polyclonal antibodies raised against the coiled-coil domain of MuRF1 (Witt et al. 2008; IgY-type avian antibody ‘avian anti-MuRF1’). In addition, we used two rabbit polyclonal antibodies raised against the RING H2 domain (’rabbit anti-RING H2 MuRF1’ and ‘rabbit anti-phospho-RING H2 MuRF1’; for more details, see www.myomedics.com). All stainings were done using standard protocols as previously described (Röder et al. 2010; Choi et al. 2012).

### Protein interaction assays (yeast two-hybrid and co-precipitations)

For yeast two-hybrid assays (Y2H), mating experiments were performed essentially as described (Witt et al. 2008). Y2H prey–bait interaction data were further verified by co-immunoprecipitations (co-IPs): for co-IP analysis, WT and MuRF1-KO mice were starved for 24 h. Mice were then killed, quadriceps muscles prepared, shock frozen in liquid nitrogen, and then stored at −80 °C. Tissues were then levigated under liquid nitrogen and homogenized in 50 mM Tris/Cl pH 8.0, 150 mM NaCl, 1 % NP40 and 10 % Glycerol, 1 mM DTT including 1× Protease- and Phosphatase-Inhibitor cocktails (Roche). After incubation at 4 °C for 1 h, extracts were centrifuged for 10 min at 4,000×*g*. Supernatants were aliquoted and frozen at −80 °C until use. For co-IP of MuRF1 and Bif-1, we combined an amount of extract corresponding to one fifth of a quadriceps in a final volume of 1 ml lysis buffer with 30 μg rabbit polyclonal anti-RING H2 MuRF1 antibody (Myomedics) and incubated with light shaking at 4 °C over night. 30 μl of Protein A-sepharose (Pierce) equilibrated in lysis buffer was added and incubated for an additional hour at 4 °C. Beads were collected by spinning 1 min at 4 °C and washed three times with 1 ml of lysis buffer. The final pellet was resuspended directly in SDS loading buffer. Affinity co-precipitation of MuRF-1 with in vivo labeled AChRs and biotinylated BGT from Invitrogen and NeutrAvidin agarose from ThermoScientific was performed as described previously (Röder et al. 2008, 2010; Choi et al. 2012). For Western blotting, samples were run on 4–12 % BisTris-Gels (Invitrogen) and blotted onto nitrocellulose membranes. Detection of MuRF1 was performed with anti-MuRF1 IgY-type antibodies from chicken (Myomedics), coupled to anti chicken-AP (Jackson). Bif-1 was detected with α-Bif-1 (goat, Abcam 1343), α-goat-biotinylated (Dako) and Streptavidin-AP (Pierce). Monoclonal antibodies against AChR α-subunit (610989) and α-actinin (A7811) were from BD Bioscience and Sigma-Aldrich, respectively. Polyclonal antibody against β1-adrenergic receptor (PA1-049) was from Affinity Bioreagents. Secondary anti-mouse and anti-rabbit antibodies coupled to HRP were from Dako.

### In vivo transfection, imaging and AChR lifetime determination

Expression of heterologous fusion proteins employed MuRF1 and Bif-1 both fused to EGFP in pEGFP-C1 (for MuRF1-eGFP-C1 fusion constructs, see McElhinny et al. 2002). Similarly, Bif-1 was inserted into pEGFP-C1. Transfection of expression constructs into tibialis anterior muscles was done as previously described (Dona et al. 2003; Röder et al. 2008). Ten days later, mice were anaesthetized and transfected muscles exposed and injected with BGT-AF647 as described previously (Röder et al. 2008; Rudolf et al. 2012). Then, mice were transferred to the confocal microscope (DMRE TCS SP2, Leica Microsystems). GFP and BGT-AF647 fluorescence were excited using a KrAr laser (488 nm) and a HeNe laser (633 nm), respectively. Emission was detected by a 63x/1.2NA HCX PL APO CS W CORR objective (Leica Microsystems) (immersion medium, Visc-Ophtal gel, Winzer-Pharma) using 500–550 and 650–750 nm bandpass settings for GFP and BGT-AF647, respectively, in the SP2 unit. Microscopic determination of AChR turnover was done as described before (Röder et al. 2008, 2010; Choi et al. 2012). In brief, infrared fluorescent BGT-AF647 (first injection, labels 'old receptor' pool) and red fluorescent BGT-AF555 (second injection, labels 'new receptor' pool) were sequentially injected at a temporal distance of 10 days. After the second injection muscles were examined in vivo with confocal microscopy. Determination of AChR lifetime was executed as recently described (Strack et al. 2011; Choi et al. 2012) and used ^125^I-labeled BGT (^125^I-BGT) from Perkin Elmer. ^125^I emission was quantified using a liquid nitrogen-cooled GX-3018 germanium semiconductor detector (Canberra) and an electrically cooled GEM-FX5825P4-S germanium semiconductor detector (Ortec).

## Results

### MuRF1 is highly enriched beneath the NMJ

**Fig. 1:**
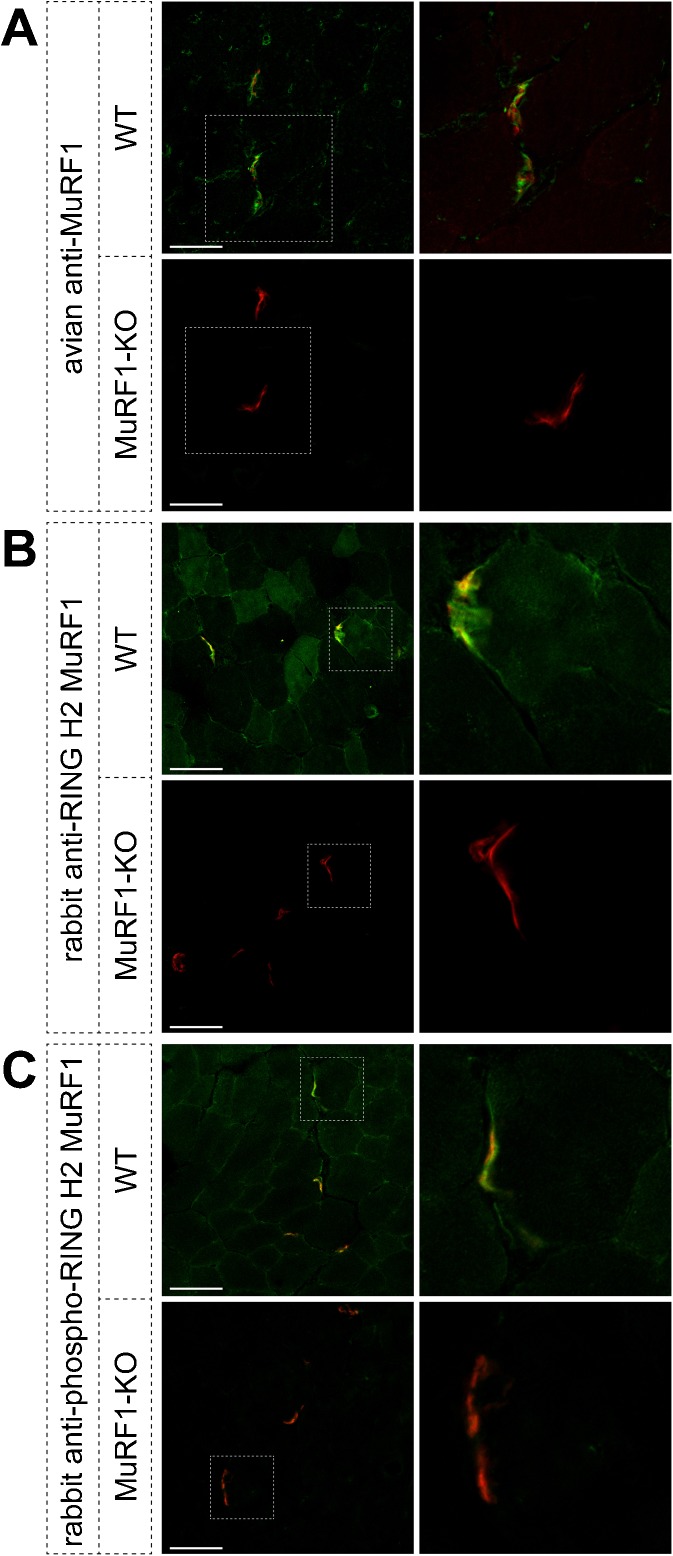
Endogenous MuRF1 is highly enriched in close proximity to the NMJ. EDL muscles of WT and MuRF1-KO animals were sectioned transversally and co-stained with the AChR marker, BGT-AF555, and with three different anti-MuRF1 antibodies, i.e., IgY-type from chicken against the MuRF1 coiled-coil domain (**a**); IgG-type rabbit polyclonal antibodies directed to the MuRF1 RING H2 domain (**b**, **c**). Fluorescence distribution was then determined using confocal microscopy. Pictures show single optical sections of fluorescence signals of BGT-AF555 (*red*) and anti-MuRF1 (*green*) of WT and MuRF1-KO muscles, as indicated. *Right panels* depict high power views of the *boxed areas* of the images on the *left*. Scale bars represent 50 μm

**Fig. 2:**
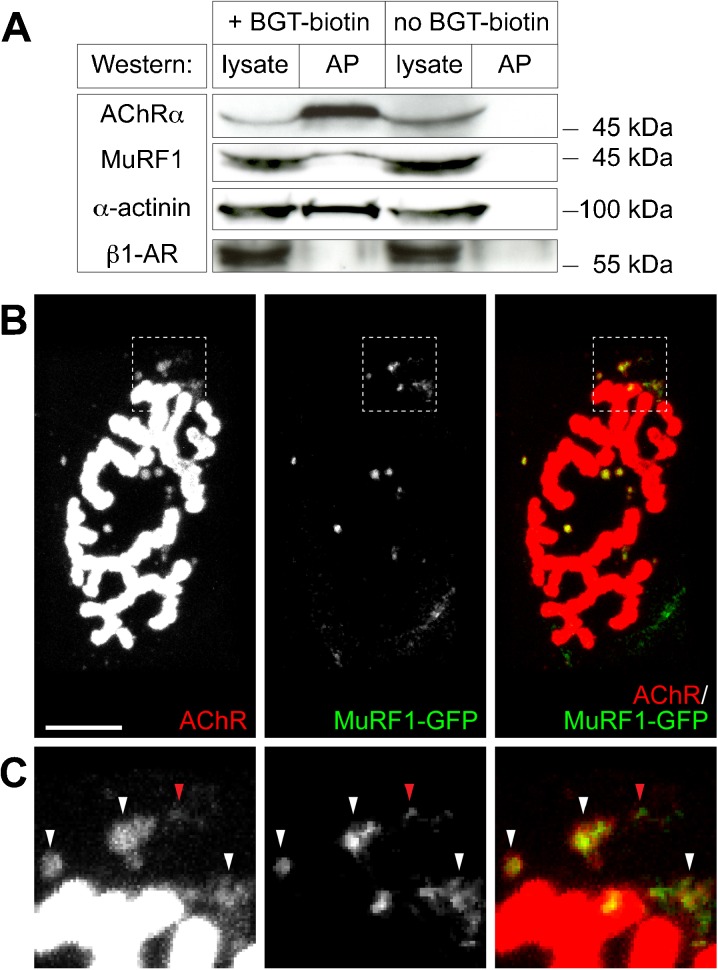
MuRF1 interacts with AChR on endo/lysosomal carriers. **a** BGT-biotin or solvent was injected into gastrocnemius muscles. Five hours later, muscles were collected and lysates prepared. From these, BGT-biotin-bound AChR and its interaction partners were affinity-precipitated with neutravidin beads. Panels show Western blot bands of lysates and affinity precipitates (*AP*) upon exposure to antibodies against AChRα, MuRF1, α-actinin (positive control), or β1-adrenergic receptor (*β1-AR*, negative control), as indicated. **b**–**c** Tibialis anterior muscles were transfected with MuRF1-GFP. Ten days later, muscles were injected with the fluorescent AChR marker, BGT-AF647, and then monitored with in vivo confocal imaging. After acquisition, all images were electronically contrasted to highlight the weak endocytic AChR signals. In overlay pictures, AChR and GFP signals appear *red* and *green*, respectively. Colocalization of AChR and GFP signals is depicted in yellow. **b** Median filtered single optical planes of fluorescence signals. Scale bar represents 20 μm. **c** Panels depict high power views of the *boxed* regions shown in **b**. *White* and *red arrowheads* indicate colocalizing and partially overlapping signals, respectively, in endocytic carriers

In previous studies, MuRF1 was found to associate with the giant myofibrillar protein titin within its M-line integral segment (Centner et al. 2001; Mrosek et al. 2007; Hirner et al. 2008). Another set of studies using different antibodies localized MuRF1 epitopes in the soluble fraction in the cytoplasm (Cohen et al. 2009). However, because MuRF1 is part of a highly conserved RING finger protein family, antibodies may cross-react with the three known members of the MuRF gene family. So far, nothing is known about a possible role of MuRF1 in the area of the NMJ. To address this issue we first investigated if MuRF1 is expressed in the NMJ region using different antibodies raised to MuRF1-specific epitopes. Muscle cross-sections were co-stained with fluorescent BGT-AF555, a marker of AChRs, and three different anti-MuRF1 antibodies, which were raised either to the coiled-coil or to the RING H2 domain of the molecule (for more details, see www.myomedics.com). Notably, sections from wild-type muscles displayed for all three anti-MuRF1 antibodies a high labeling intensity in close proximity of the NMJs and much weaker signals in the sarcomeric regions (Fig. 1). Conversely, sections from MuRF1-KO animals were almost completely devoid of immunofluorescence signals (Fig. 1). This shows that MuRF1 accumulates in the endplate areas of skeletal muscle fibers suggesting a specific role of this protein in synapse-related processes. This localization was consistently detected with the three different types of anti-MuRF1 antibodies. To further address the possibility of cross-reactivity with the other highly related RING-finger proteins MuRF2 or MuRF3, we also analyzed the targeting of MuRF1-GFP fusion proteins in vivo in transgenic muscles (see section on MuRF1-GFPs; Fig. 2b and c). These experiments confirmed the targeting of MuRF1 to the NMJ.

### MuRF1 co-precipitates with AChRs

Prompted by the unexpectedly strong subsynaptic enrichment of MuRF1 we then tested the association of the E3 ligase with synaptic components. The major postsynaptic protein of the NMJ is the AChR, which is first exposed at the postsynaptic membrane and then eventually undergoes cycles of endocytosis and lysosomal degradation or recycling, in an activity-dependent manner (Engel et al. 1977; Stanley and Drachman 1981 Fumagalli et al. 1982; Akaaboune et al. 1999; Bruneau et al. 2005). To screen for a potential interaction between MuRF1 and AChR we first used a previously established assay based on the α-bungarotoxin- (BGT) mediated affinity precipitation of cell surface-exposed and endocytosed AChRs in vivo (Röder et al. 2008). The 8-kDa snake venom, BGT, is not membrane-permeable and binds to the extracellular part of AChRs with extremely high specificity and in an essentially irreversible manner (Changeux et al. 1970; Akaaboune et al. 1999). We injected BGT-biotin into live gastrocnemius muscle, allowing it to first bind to surface-exposed AChRs and then to follow the AChRs during endocytosis and recycling. Few hours later, muscles were harvested and lysates prepared. BGT-biotin-bound AChRs were then precipitated using standard protocols and lysates and affinity precipitates analyzed by SDS-PAGE and Western blots (Fig. 2a). This showed a robust precipitation of AChRs and of the AChR-interacting protein, α-actinin. While β1-adrenergic receptor, serving as a negative control, did not precipitate, MuRF1 was always present in the sediments, although it was found there in varying amounts. Figure 2a shows a representative experiment, but stronger MuRF1 bands in the precipitate were also observed. Furthermore, control preparations run without BGT-biotin injection were all negative, hence strongly suggesting an interaction between MuRF1 and AChR.

### MuRF1-GFP colocalizes with endocytic AChRs

Next, to investigate if the in vitro biochemical interaction between AChRs and MuRF1 relates to their localization in vivo, we transfected MuRF1-GFP fusion constructs into live tibialis anterior muscles. Ten days after transfection, NMJs were labeled with fluorescent BGT-AF647 and about an hour later muscles were imaged in situ at the confocal microscope. In general, MuRF1-GFP accumulated in the immediate vicinity of the NMJs in small, punctuated structures (Fig. 2b). Notably, nearly all of these structures co-localized with puncta positive for endocytic AChRs (Fig. 2b–c, white arrowheads in c). In a few cases, MuRF1-GFP and BGT-AF647 signals were not perfectly overlapping but very close to each other (Fig. 2c, red arrowhead). These results corroborate an interaction of MuRF1 with AChR and indicate that this interaction occurs mainly on carriers containing AChRs in an endocytic, recycling or lysosomal compartment.

### MuRF1 does not affect AChR stability under non-challenged basal conditions

**Fig. 3:**
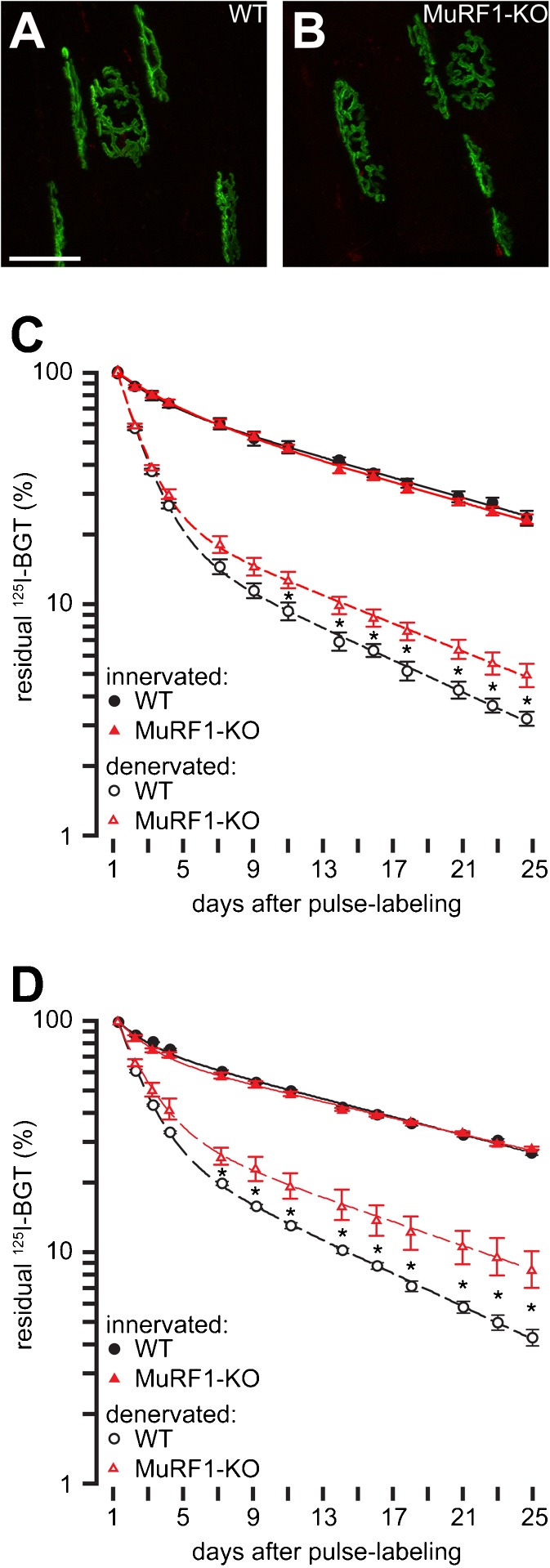
Metabolic destabilization of AChR upon denervation is partially rescued in MuRF-1KO animals. **a** Tibialis anterior muscles of wild-type (*WT*) and **b** MuRF1-KO animals were injected with infrared fluorescent BGT-AF647 to label AChRs present at that time point ('old receptors'). Ten days later, red fluorescent BGT-AF555 was injected to mark 'new receptors' and then muscles were imaged with confocal microscopy. Panels show maximum-z projections of 'old receptors' and 'new receptors' in *green* and *red*, respectively. Note, that all NMJs displayed hardly any 'new receptor' signals. Left legs of WT and MuRF1-KO animals were denervated, right legs served as innervated controls (**c**) or were left untreated and innervated controls were from separate animals (**d**). Five days later, AChRs in tibialis anterior muscles of all legs were pulse-labeled with ^125^I-BGT. Then, ^125^I emission was monitored at indicated intervals during the next 4 weeks and normalized to the values measured 24 h after pulse labeling. Symbols show measured values (mean ± SEM, **c**: *n* = 3 for WT and *n* = 4 for MuRF1-KO, **d**: *n* = 4 for both denervated WT and denervated MuRF1-KO muscles and *n* = 5 for both innervated WT and innervated MuRF1-KO muscles). *Lines* depict two-term exponential fits. *p < 0.05

Given the potential interaction between MuRF1 and AChR on one hand, and the well-established roles of ubiquitination on lysosomal targeting of membrane receptors (Haglund and Dikic 2012) and the maintenance of the NMJ (Lu et al. 2007) on the other hand, we then studied if MuRF1 has a functional impact on AChR turnover. First, we employed a previously established in vivo imaging approach to study NMJ morphology and AChR stability in MuRF1-KO mice (Röder et al. 2008, 2010). In this assay, sequential pulse labeling of AChR pools with differentially colored BGT species indicates the intensity of AChR turnover. A first injection indelibly marks 'old receptors' with infrared fluorescent BGT-AlexaFluor647. Ten days later a second application of BGT, tagged with red fluorescent AlexaFluor555, stains 'new receptors' that were not present during the BGT-AlexaFluor647 injection. Under normal conditions with this procedure, most AChRs will carry the ‘old receptor’ stamp (BGT-AF647; green color in Fig. 3a–b) and only very few show the ‘new receptor’ label (BGT-AF555; red color in Fig. 3a–b). Interestingly, using this approach, both, wild-type (Fig. 3a; WT) and MuRF1-KO muscles (Fig. 3b) displayed completely normal NMJ morphology and apparent AChR turnover, suggesting that MuRF1 does not influence AChR stability under normal trophic conditions.

### Under atrophic conditions AChR lifetime reduction is partially rescued in MuRF1-KO animals

To further explore the involvement of MuRF1 in AChR stability, we used a recently developed AChR radio-labeling assay (Strack et al. 2011), where AChRs are pulse-labeled with radioactive ^125^I-BGT and ^125^I emission is then followed over 4 weeks. Applying this procedure to wild-type and MuRF1-KO animals confirmed the in vivo-imaging result that under normal trophic conditions MuRF1 plays no role in AChR lifetime regulation (Fig. 3c–d, solid lines). Since MuRF1 has been postulated to function as an atrogene, i.e., as being activated under atrophic conditions and to promote sarcomeric loss, we then moved to an atrophy-inducing condition, in this case denervation. Therefore, muscles were surgically denervated at the level of the sciatic nerve 5 days before the ^125^I-BGT pulse. Denervation led to a marked reduction in the half-life of AChRs and it did so in both, wild-type as well as MuRF1-KO animals (Fig. 3c–d, dashed lines). However, the AChR lifetime reduction was significantly smaller in MuRF1-KO (Fig. 3c–d). This shows that MuRF1 plays a pivotal role in activity-dependent AChR turnover.

### AChR stabilization under denervation-induced atrophy is mediated by MuRF1 but not MuRF2

**Fig. 4:**
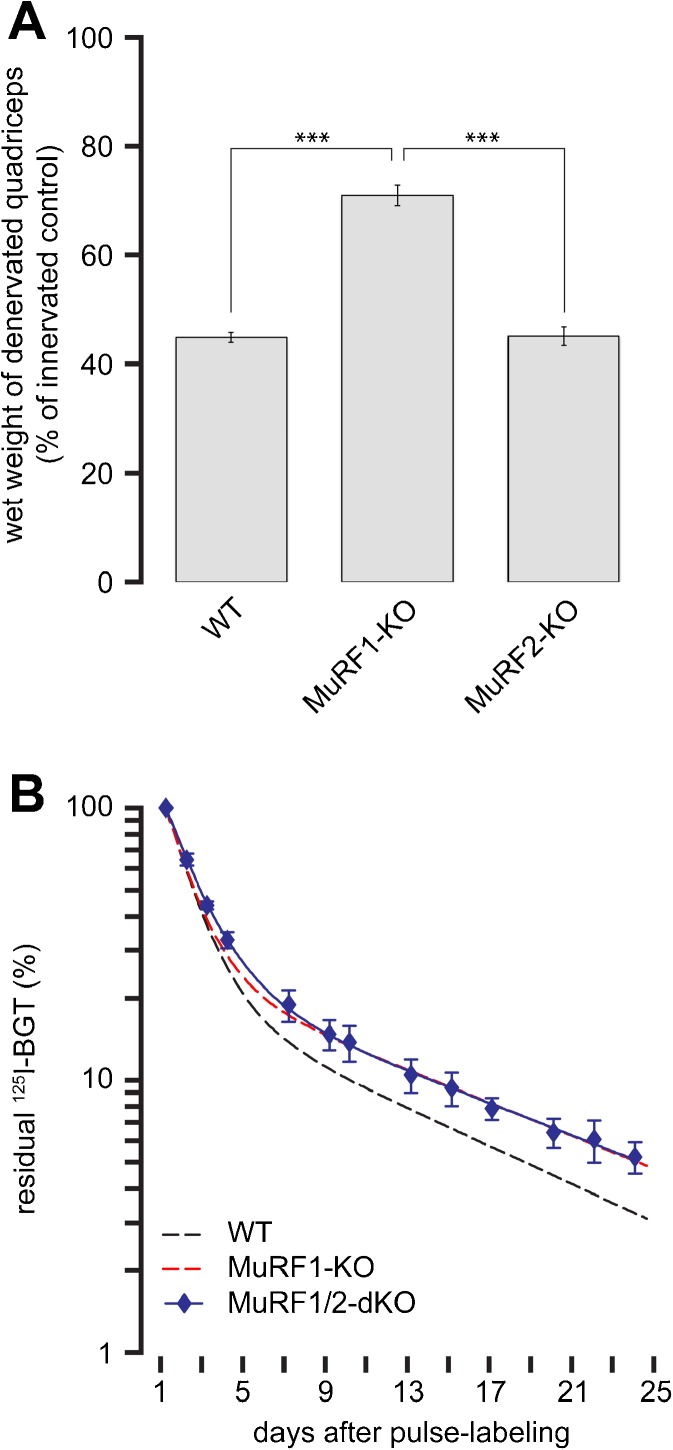
MuRF1 but not MuRF2 regulates skeletal trophicity and innervation. **a** Muscle masses of whole quadriceps muscles were monitored 14 days after denervation in MuRF1- and MuRF2-KO mice. Inactivation of MuRF1 but not MuRF2 protects partially from muscle mass loss consistent with earlier data. Columns show mean ± SEM (*n* = 7) *p < 0.001 according to Welch test. **b** MuRF2 inactivation does not alter AChR stability. Five days after sciatic denervation, AChRs in tibialis anterior muscles were pulse-labeled with ^125^I-BGT. ^125^I emission was monitored at indicated intervals during the next 4 weeks and normalized to the values measured 24 h after pulse labeling. Symbols show measured values (mean ± SEM, *n* = 2 for MuRF1/2-dKO). *Lines* depict two-term exponential fits, WT and MuRF1-KO fits, see Fig. 3b

Previous studies have revealed a cooperative function of MuRF1 and MuRF2 for maintaining muscle mass under normal conditions (Witt et al. 2008). To find out if such cooperation is also active upon catabolic stimulation, we first determined muscle mass following denervation in wild-type (WT) and MuRF1- or MuRF2-deficient mice. Although denervation induced significant atrophy in all three strains, muscles lacking MuRF1 were least affected and lost only about 30 % of their wet weight within 2 weeks (Fig. 4a). Conversely, muscles from MuRF2-KO animals were indistinguishable from WT and lost more than 50 % of wet weight (Fig. 4a). This shows that MuRF1 is a major determinant of muscle catabolism, while MuRF2 plays apparently no role in this process. The observation of a MuRF1-specific regulation of muscle atrophy upon denervation prompted us to investigate AChR stability in mice lacking MuRF2. Thus, we employed the radioiodine assay to determine AChR lifetimes in MuRF1/2-double KO. Notably, these animals did not exhibit any enhancement of AChR stability upon denervation as compared to MuRF1-KO (Fig. 4b). Therefore, the partial rescue of AChR lifetime upon denervation was MuRF1-specific and did not involve MuRF2. These data further consolidate a primary function of MuRF1 in muscle catabolism.

### Endophilin B1/Bif-1 interacts with MuRF1 in an isoform-specific manner

**Fig. 5:**
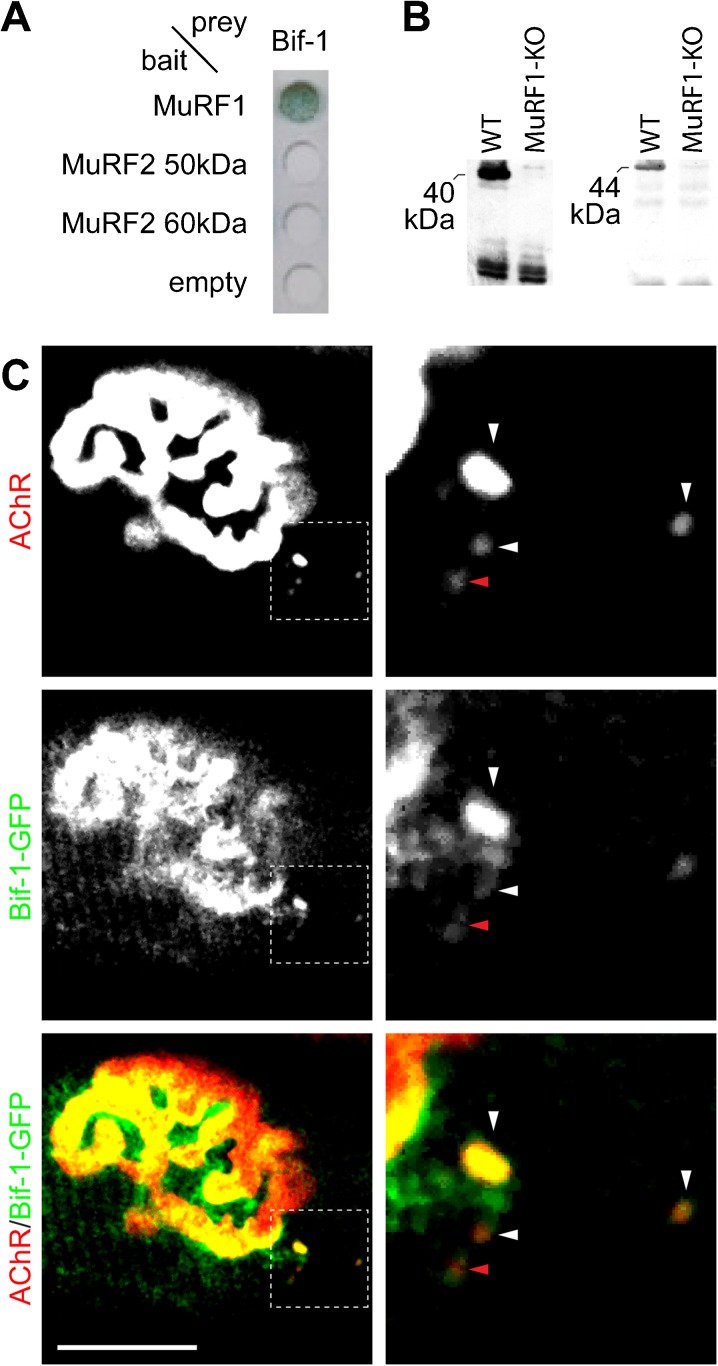
Bif-1 interacts with MuRF1 and targets to the NMJ and endo/lysosomal carriers containing AChR. **a** Bif-1 was found to interact with MuRF1 in a previous yeast two-hybrid screen (Witt et al. 2008). Follow-up by mating confirms this interaction and moreover suggests that this interaction is specific for MuRF1 in the MuRF protein RING finger family, since mating with MuRF2 did not yield β-galactosidase reporter activity (*blue*). **b** MuRF1 and Bif-1 form a complex in muscle extracts as indicated by pulldown: MuRF1 and its associated binding proteins were precipitated with a MuRF1 specific antibody from soluble TA extracts. In WT muscle extracts, MuRF1 and Bif-1 immunopositive signals were detected with specific antibodies in the pull-down at 40 and 44 kDa, respectively. Both signals were absent in precipitates from MuRF1-KO muscle. **c** Tibialis anterior muscles were transfected with Bif-1-GFP. Ten days later, muscles were injected with the fluorescent AChR marker, BGT-AF647, and then monitored with in vivo confocal imaging. *Left panels* show median filtered single optical planes of fluorescence signals. *Right panels* depict high power views of the boxed regions in *left panels*. After acquisition, all images were electronically contrasted to highlight the weak endocytic AChR signals. In overlay pictures, AChR and GFP signals appear *red* and *green*, respectively. Colocalization of AChR and GFP signals are shown in *yellow*. Scale bar represents 20 μm

The finding of a MuRF1-exclusive role in muscle catabolism and its apparent involvement in AChR turnover led us to hypothesize that MuRF1 may act in the regulation of endo/lysosomal trafficking. To investigate this further, we re-surveyed prey clones from a previously published yeast-two-hybrid screen (Witt et al. 2008) for potential links to endo/lysosomal trafficking. As a possible candidate, we identified a prey clone coding for endophilin B1 alias Bif-1 (Bax-interacting factor-1). Endophilin B1/Bif-1 was originally found to orchestrate autophagy, mitochondrial morphology, and apoptosis (Takahashi et al. 2009). A more recent study identified this protein as crucial for the metabolic turnover of nerve growth factor receptor, TrkA (Wan et al. 2008; Cheung and Ip 2009). Notably, mating experiments demonstrated that Bif-1 interacts with MuRF1, but not with MuRF2 (Fig. 5a). To further consolidate the interaction between Bif-1 and MuRF1 we performed co-IP. Lysates were prepared from quadriceps muscles, and MuRF1 was precipitated using a rabbit polyclonal antiserum raised to the MuRF1 RING H2 domain. Western blot showed a Bif-1-positive band in the precipitate at 44 kDa, which was absent in MuRF1-KO preparations (Fig. 5b). Together, these data show that Bif-1 is a novel interaction partner of MuRF1 and that within the MuRF1, 2, 3 family this interaction is specific for the MuRF1 isoform.

### Bif-1-GFP is enriched at the NMJ and colocalizes with endocytic AChRs

Finally, we asked if Bif-1 could potentially mediate the linkage of MuRF1 to the endo/lysosomal compartment containing AChRs. To address this, we studied the subcellular distribution of Bif-1 by expressing a Bif-1-GFP fusion protein in live mouse tibialis anterior muscle. NMJs were labeled with fluorescent BGT-AF647 and fluorescence signals were then monitored with in vivo imaging. This showed an interesting distribution pattern of Bif-1-GFP (Fig. 5c): While a minor portion of the GFP fluorescence was observed in a striated pattern of the sarcomeric region, large amounts of fusion protein were detected in the NMJ region. Additionally, punctuate structures were found, resembling those of MuRF1-GFP in number and localization next to the NMJ. Interestingly, these puncta again largely colocalized with or mounted carriers containing endocytosed AChRs (Fig. 5c, white or red arrowheads, respectively). This suggests that Bif-1 might serve a similar function for AChR turnover as for TrkA.

## Discussion

Catabolic stress conditions induce a conserved set of transcripts during muscle wasting (referred to as atrogenes) thus holding promise to possibly attenuate this genetic reprogramming (Bodine et al. 2001). These atrogenes include several ubiquitin E3 ligases, enzymes that initiate the degradation of their target proteins by catalyzing the addition of ubiquitin to exposed lysine residues in their respective targets in conjunction with non-rate limiting E1 and E2 ligase activities. Thereby, induction of atrophy-associated E3 ligases is thought to induce muscle atrophy by the degradation of sarcomeric muscle proteins, including in particular myosin, actin, and troponin I (Bodine et al. 2001; Glass 2005, 2010; Adams et al. 2008).

### Sarcomeric and NMJ remodeling are co-regulated by MuRF1

Here, we have tested the hypothesis that during muscle atrophy, E3 ligase-driven wasting of sarcomeres and NMJ-driven remodeling could be linked together: Recent studies suggest an important involvement of the NMJ, since genetic subversion of NMJ stability leads to major signs of aging-related atrophy, including fiber type grouping, fiber size heterogeneity, and fiber loss (Butikofer et al. 2011). Therefore, functional muscle fiber denervation is a major reason for aging-related atrophy (Rowan et al. 2012). Furthermore, the finding of NMJ denervation without motoneuron loss in aging mice shows that, at least partially, muscle-specific processes can lead to functional denervation in aging-related atrophy (Chai et al. 2011). Because Rowan et al. (2012) found that protein expression of the E3 ligase MuRF1 (an atrogene that has been extensively studied in sarcomeric muscle protein degradation) was highest in denervated muscle fibers, we concentrated here on the role of MuRF1 in the NMJ. Consistent with our hypothesis that E3 ligases such as MuRF1 may link together sarcomeric and NMJ remodeling, we found that MuRF1 was highly enriched in the NMJ region: MuRF1 immunostaining obtained with three distinct anti-MuRF1 antibodies was much stronger in the NMJ regions than in the rest of the muscle fibers (Fig. 1). This raises the possibility of a specific yet undiscovered function of MuRF1 at the synapse.

### MuRF1 plays a role in NMJ control by regulating AChR lifetime

Given the presence of MuRF1 in the NMJ we tested then if MuRF1 might participate in synaptic functioning of the major player in the postsynaptic portion of the NMJ, i.e., the AChR. This ligand-gated pentameric ion channel (Karlin 2002) transduces motorneuronal activity into skeletal muscle activity and thus mediates any kind of voluntary muscle contraction (Martyn et al. 2009). Normally, AChR occurs at a quasi-crystalline density of roughly 10,000 receptors per square micron on the postsynaptic membrane (Fambrough 1979; Sanes and Lichtman 2001) and mutations of the receptor, autoimmune responses against it, or factors that reduce its amount are causative for severe muscle weakness and diseases like myasthenia gravis or congenital myasthenic syndromes (Engel et al. 1977; Ohno and Engel 2002; Müller et al. 2007; Palace and Beeson 2008). Co-IPs suggested that MuRF1 could interact directly with AChR (Fig. 2). Importantly, we also found that MuRF1 controls the metabolic lifetime of AChRs under atrophying conditions (Fig. 3). Given that AChR lifetime is known to vary with AChR transcriptional activity in denervated muscles (Yampolsky et al. 2010b) and that MuRF1 can also be involved in the regulation of protein biosynthesis (Witt et al. 2008), further studies are required to determine putative contributions of MuRF1 to the biogenesis of AChRs. Our data imply that MuRF1 fulfills at least one more, completely novel function, i.e., the regulation of the skeletal muscle innervation status.

### MuRF1 likely acts on regulating endo/lysosomal progression of AChR

Mechanistically, the regulation of the metabolic stability of the AChR is tightly coupled to its activity (Levitt et al. 1980; Loring and Salpeter 1980; Levitt and Salpeter 1981; Stanley and Drachman 1981, 1983; Shyng et al. 1991; Strack et al. 2011), and this largely involves vesicular transport processes (Fumagalli et al. 1982; Akaaboune et al. 1999). Indeed, like any other typical transmembrane protein, newly generated AChRs reach the postsynapse in exocytic carriers (Marchand et al. 2000, 2002; Marchand and Cartaud 2002). Subsequently, AChRs get endocytosed (Fumagalli et al. 1982; Akaaboune et al. 1999) and either degraded in lysosomes (Clementi et al. 1983; Kumari et al. 2008; Valkova et al. 2011) or recycled (Akaaboune et al. 1999; Bruneau et al. 2005); this decision is clearly muscle activity-dependent and the recycling process involves the cooperative function of myosin Va, protein kinase A, and the anchor protein, rapsyn (Röder et al. 2008, 2010; Yampolsky et al. 2010a; Choi et al. 2012). For many other receptor molecules a conserved cascade of small Rab-GTPases is instrumental in the progression from endosomes to recycling endosomes or lysosomes (Huotari and Helenius 2011). In particular, the exchange of Rab5 with Rab7 appears to be critical for the endo/lysosomal maturation. Much less is known about the AChR degradation route, and our data implicate MuRF1 here. Consistent with this hypothesis, MuRF1 is downregulated during physical activity (Adams et al. 2008), and, conversely, it is strongly upregulated during physical inactivity. Thus, the rapsyn- and protein kinase A activity-promoted stabilization on the one hand and the inactivity-promoted MuRF1-dependent destabilization of AChRs on the other hand are likely to be physiologically antagonistic regulatory processes. How MuRF1 exerts this regulatory function mechanistically is unclear, but it is likely that it involves further factors. In this study we identified one such candidate molecule, Bif-1 alias endophilin B1. Recently, this liposome tubulation factor was found to localize on early endosomes and to regulate the endo/lysosomal progression of the nerve growth factor receptor, TrkA (Wan et al. 2008; Cheung and Ip 2009). Thus, we were excited to find that MuRF1 interacts with both AChR (Fig. 2) and Bif-1 (Fig. 5). Furthermore, MuRF1 and Bif-1 both colocalize with endocytic AChRs in close proximity to the NMJ (Figs. 2 and 5). These observations strongly suggest that Bif-1 might play a similar role in endo/lysosomal progression for AChRs as for TrkA.

### Outlook — MuRF1-specific activation signals needed?

Notably, the determination of AChR lifetime kinetics of MuRF1-KO animals under normal and atrophic (denervation) regimes demonstrates that MuRF1 plays a pivotal role in AChR degradation only during atrophy but not under standard trophic conditions (Fig. 3): a significantly reduced AChR destabilization develops only upon denervation. This observation is consistent with the finding that overexpression of MuRF1 alone without additional signals is not sufficient to trigger muscle wasting (Hirner et al. 2008), raising the possibility that atrogene function(s) of MuRF1 may require specific atrophic stimuli for activation. In this context, we speculate that the sarcomere-associated form of MuRF1 observed in the M-line after overexpression in healthy non-challenged myocytes (McElhinny et al. 2002; Hirner et al. 2008) represents a titin-based complex that still requires an activation signal for the translocation of MuRF1 into the cytoplasm and/or the NMJ. Finally, our studies on AChR lifetime regulation demonstrate a specific role for MuRF1, since, first, inactivation of MuRF2 alone is not sufficient to attenuate wasting (Fig. 4a), second, AChR lifetime kinetics in MuRF1,2-double-KO animals were identical to MuRF1-single-KO animals (Fig. 4b), and, third, the interaction with Bif-1 was specific for MuRF1 within the MuRF family (Fig. 5). Taken together, our data implicate MuRF1 as a key molecular regulator in stimulated atrophy processes that cannot be compensated for by MuRF2 or MuRF3. Future studies will need to clarify if specific factors can activate MuRF1, and how activated MuRF1 and Bif-1 may cooperate during their joint functioning on sarcomeric and NMJ remodeling.

## References

[CR1] Adams V, Mangner N, Gasch A, Krohne C, Gielen S, Hirner S, Thierse HJ, Witt CC, Linke A, Schuler G, Labeit S (2008). Induction of MuRF1 is essential for TNF-alpha-induced loss of muscle function in mice. J Mol Biol.

[CR2] Akaaboune M, Culican SM, Turney SG, Lichtman JW (1999). Rapid and reversible effects of activity on acetylcholine receptor density at the neuromuscular junction in vivo. Science.

[CR3] Andonian MH, Fahim MA (1987) Effects of endurance exercise on the morphology of mouse neuromuscular junctions during ageing. J Neurocytol 16:589–599 10.1007/BF01637652 3694234

[CR4] Andonian MH, Fahim MA (1988) Endurance exercise alters the morphology of fast- and slow-twitch rat neuromuscular junctions. Int J Sports Med 9:218–223 10.1055/s-2007-1025009 3410628

[CR5] Bodine SC, Latres E, Baumhueter S, Lai VK, Nunez L, Clarke BA, Poueymirou WT, Panaro FJ, Na E, Dharmarajan K, Pan ZQ, Valenzuela DM, DeChiara TM, Stitt TN, Yancopoulos GD, Glass DJ (2001). Identification of ubiquitin ligases required for skeletal muscle atrophy. Science.

[CR6] Bruneau E, Sutter D, Hume RI, Akaaboune M (2005). Identification of nicotinic acetylcholine receptor recycling and its role in maintaining receptor density at the neuromuscular junction in vivo. J Neurosci.

[CR7] Butikofer L, Zurlinden A, Bolliger MF, Kunz B, Sonderegger P (2011). Destabilization of the neuromuscular junction by proteolytic cleavage of agrin results in precocious sarcopenia. FASEB J.

[CR8] Centner T, Yano J, Kimura E, McElhinny AS, Pelin K, Witt CC, Bang ML, Trombitas K, Granzier H, Gregorio CC, Sorimachi H, Labeit S (2001). Identification of muscle specific ring finger proteins as potential regulators of the titin kinase domain. J Mol Biol.

[CR9] Chai RJ, Vukovic J, Dunlop S, Grounds MD, Shavlakadze T (2011). Striking denervation of neuromuscular junctions without lumbar motoneuron loss in geriatric mouse muscle. PLoS One.

[CR10] Changeux JP, Kasai M, Lee CY (1970). Use of a snake venom toxin to characterize the cholinergic receptor protein. Proc Natl Acad Sci U S A.

[CR11] Chen J, Mizushige T, Nishimune H (2012). Active zone density is conserved during synaptic growth but impaired in aged mice. J Comp Neurol.

[CR12] Cheung ZH, Ip NY (2009). Endophilin B1: guarding the gate to destruction. Commun Integr Biol.

[CR13] Choi KR, Berrera M, Reischl M, Strack S, Albrizio M, Röder IV, Wagner A, Petersen Y, Hafner M, Zaccolo M, Rudolf R (2012). Rapsyn mediates subsynaptic anchoring of PKA type I and stabilisation of acetylcholine receptor in vivo. J Cell Sci.

[CR14] Clementi F, Sher E, Erroi A (1983). Acetylcholine receptor degradation: study of mechanism of action of inhibitory drugs. Eur J Cell Biol.

[CR15] Cohen S, Brault JJ, Gygi SP, Glass DJ, Valenzuela DM, Gartner C, Latres E, Goldberg AL (2009). During muscle atrophy, thick, but not thin, filament components are degraded by MuRF1-dependent ubiquitylation. J Cell Biol.

[CR16] Courtney J, Steinbach JH (1981). Age changes in neuromuscular junction morphology and acetylcholine receptor distribution on rat skeletal muscle fibres. J Physiol.

[CR17] Dona M, Sandri M, Rossini K, Dell'Aica I, Podhorska-Okolow M, Carraro U (2003). Functional in vivo gene transfer into the myofibers of adult skeletal muscle. Biochem Biophys Res Commun.

[CR18] Engel AG, Lindstrom JM, Lambert EH, Lennon VA (1977). Ultrastructural localization of the acetylcholine receptor in myasthenia gravis and in its experimental autoimmune model. Neurology.

[CR19] Fambrough DM (1979). Control of acetylcholine receptors in skeletal muscle. Physiol Rev.

[CR20] Fumagalli G, Engel AG, Lindstrom J (1982). Ultrastructural aspects of acetylcholine receptor turnover at the normal end-plate and in autoimmune myasthenia gravis. J Neuropathol Exp Neurol.

[CR21] Glass DJ (2005). Skeletal muscle hypertrophy and atrophy signaling pathways. Int J Biochem Cell Biol.

[CR22] Glass DJ (2010). Signaling pathways perturbing muscle mass. Curr Opin Clin Nutr Metab Care.

[CR23] Gutmann E, Hanzlikova V (1976). Fast and slow motor units in ageing. Gerontology.

[CR24] Haglund K, Dikic I (2012). The role of ubiquitylation in receptor endocytosis and endosomal sorting. J Cell Sci.

[CR25] Hirner S, Krohne C, Schuster A, Hoffmann S, Witt S, Erber R, Sticht C, Gasch A, Labeit S, Labeit D (2008). MuRF1-dependent regulation of systemic carbohydrate metabolism as revealed from transgenic mouse studies. J Mol Biol.

[CR26] Huotari J, Helenius A (2011). Endosome maturation. EMBO J.

[CR27] Karlin A (2002). Emerging structure of the nicotinic acetylcholine receptors. Nat Rev Neurosci.

[CR28] Kumari S, Borroni V, Chaudhry A, Chanda B, Massol R, Mayor S, Barrantes FJ (2008). Nicotinic acetylcholine receptor is internalized via a Rac-dependent, dynamin-independent endocytic pathway. J Cell Biol.

[CR29] Latronico N, Peli E, Botteri M (2005). Critical illness myopathy and neuropathy. Curr Opin Crit Care.

[CR30] Latronico N, Shehu I, Seghelini E (2005). Neuromuscular sequelae of critical illness. Curr Opin Crit Care.

[CR31] Lecker SH, Jagoe RT, Gilbert A, Gomes M, Baracos V, Bailey J, Price SR, Mitch WE, Goldberg AL (2004). Multiple types of skeletal muscle atrophy involve a common program of changes in gene expression. FASEB J.

[CR32] Levitt TA, Salpeter MM (1981). Denervated endplates have a dual population of junctional acetylcholine receptors. Nature.

[CR33] Levitt TA, Loring RH, Salpeter MM (1980). Neuronal control of acetylcholine receptor turnover rate at a vertebrate neuromuscular junction. Science.

[CR34] Loring RH, Salpeter MM (1980). Denervation increases turnover rate of junctional acetylcholine receptors. Proc Natl Acad Sci U S A.

[CR35] Lu Z, Je HS, Young P, Gross J, Lu B, Feng G (2007). Regulation of synaptic growth and maturation by a synapse-associated E3 ubiquitin ligase at the neuromuscular junction. J Cell Biol.

[CR36] Marchand S, Cartaud J (2002). Targeted trafficking of neurotransmitter receptors to synaptic sites. Mol Neurobiol.

[CR37] Marchand S, Bignami F, Stetzkowski-Marden F, Cartaud J (2000). The myristoylated protein rapsyn is cotargeted with the nicotinic acetylcholine receptor to the postsynaptic membrane via the exocytic pathway. J Neurosci.

[CR38] Marchand S, Devillers-Thiery A, Pons S, Changeux JP, Cartaud J (2002). Rapsyn escorts the nicotinic acetylcholine receptor along the exocytic pathway via association with lipid rafts. J Neurosci.

[CR39] Martyn JA, Fagerlund MJ, Eriksson LI (2009). Basic principles of neuromuscular transmission. Anaesthesia.

[CR40] McElhinny AS, Kakinuma K, Sorimachi H, Labeit S, Gregorio CC (2002). Muscle-specific RING finger-1 interacts with titin to regulate sarcomeric M-line and thick filament structure and may have nuclear functions via its interaction with glucocorticoid modulatory element binding protein-1. J Cell Biol.

[CR41] Mrosek M, Labeit D, Witt S, Heerklotz H, von Castelmur E, Labeit S, Mayans O (2007). Molecular determinants for the recruitment of the ubiquitin-ligase MuRF-1 onto M-line titin. FASEB J.

[CR42] Müller JS, Mihaylova V, Abicht A, Lochmüller H (2007). Congenital myasthenic syndromes: spotlight on genetic defects of neuromuscular transmission. Expert Rev Mol Med.

[CR43] Ohno K, Engel AG (2002). Congenital myasthenic syndromes: genetic defects of the neuromuscular junction. Curr Neurol Neurosci Rep.

[CR44] Palace J, Beeson D (2008). The congenital myasthenic syndromes. J Neuroimmunol.

[CR45] Pestronk A, Drachman DB, Griffin JW (1980). Effects of aging on nerve sprouting and regeneration. Exp Neurol.

[CR46] Röder IV, Petersen Y, Choi KR, Witzemann V, Hammer JA, Rudolf R (2008). Role of Myosin va in the plasticity of the vertebrate neuromuscular junction in vivo. PLoS One.

[CR47] Röder IV, Choi KR, Reischl M, Petersen Y, Diefenbacher ME, Zaccolo M, Pozzan T, Rudolf R (2010). Myosin Va cooperates with PKA RIalpha to mediate maintenance of the endplate in vivo. Proc Natl Acad Sci U S A.

[CR48] Rowan SL, Rygiel K, Purves-Smith FM, Solbak NM, Turnbull DM, Hepple RT (2012). Denervation causes fiber atrophy and myosin heavy chain co-expression in senescent skeletal muscle. PLoS One.

[CR49] Rudolf R, Hafner M, Mongillo M (2012). Investigating second messenger signaling in vivo. Methods Enzymol.

[CR50] Sandri M, Sandri C, Gilbert A, Skurk C, Calabria E, Picard A, Walsh K, Schiaffino S, Lecker SH, Goldberg AL (2004). Foxo transcription factors induce the atrophy-related ubiquitin ligase atrogin-1 and cause skeletal muscle atrophy. Cell.

[CR51] Sanes JR, Lichtman JW (2001). Induction, assembly, maturation and maintenance of a postsynaptic apparatus. Nat Rev Neurosci.

[CR52] Shyng SL, Xu R, Salpeter MM (1991). Cyclic AMP stabilizes the degradation of original junctional acetylcholine receptors in denervated muscle. Neuron.

[CR53] Stanley EF, Drachman DB (1981). Denervation accelerates the degradation of junctional acetylcholine receptors. Exp Neurol.

[CR54] Stanley EF, Drachman DB (1983). Rapid degradation of "new" acetylcholine receptors at neuromuscular junctions. Science.

[CR55] Strack S, Petersen Y, Wagner A, Röder IV, Albrizio M, Reischl M, Wacker IU, Wilhelm C, Rudolf R (2011). A novel labeling approach identifies three stability levels of acetylcholine receptors in the mouse neuromuscular junction in vivo. PLoS One.

[CR56] Takahashi Y, Meyerkord CL, Wang HG (2009). Bif-1/endophilin B1: a candidate for crescent driving force in autophagy. Cell Death Differ.

[CR57] Tuffery AR (1971). Growth and degeneration of motor end-plates in normal cat hind limb muscles. J Anat.

[CR58] Valdez G, Tapia JC, Kang H, Clemenson GD, Gage FH, Lichtman JW, Sanes JR (2010). Attenuation of age-related changes in mouse neuromuscular synapses by caloric restriction and exercise. Proc Natl Acad Sci U S A.

[CR59] Valkova C, Albrizio M, Röder IV, Schwake M, Betto R, Rudolf R, Kaether C (2011). Sorting receptor Rer1 controls surface expression of muscle acetylcholine receptors by ER retention of unassembled alpha-subunits. Proc Natl Acad Sci U S A.

[CR60] Wan J, Cheung AY, Fu WY, Wu C, Zhang M, Mobley WC, Cheung ZH, Ip NY (2008). Endophilin B1 as a novel regulator of nerve growth factor/TrkA trafficking and neurite outgrowth. J Neurosci.

[CR61] Witt CC, Witt SH, Lerche S, Labeit D, Back W, Labeit S (2008). Cooperative control of striated muscle mass and metabolism by MuRF1 and MuRF2. EMBO J.

[CR62] Yampolsky P, Pacifici PG, Lomb L, Giese G, Rudolf R, Röder IV, Witzemann V (2010). Time lapse in vivo visualization of developmental stabilization of synaptic receptors at neuromuscular junctions. J Biol Chem.

[CR63] Yampolsky P, Pacifici PG, Witzemann V (2010). Differential muscle-driven synaptic remodelin in the neuromuscular junction after denervation. Eur J Neurosci.

